# Early Microglial Changes Associated with Diabetic Retinopathy in Rats with Streptozotocin-Induced Diabetes

**DOI:** 10.1155/2021/4920937

**Published:** 2021-12-08

**Authors:** Young Gun Park, Ji-Yeon Lee, Chongtae Kim, Young-Hoon Park

**Affiliations:** ^1^Department of Ophthalmology and Visual Science, Seoul St. Mary's Hospital, College of Medicine, The Catholic University of Korea, Seoul, Republic of Korea; ^2^Catholic Institute for Visual Science, College of Medicine, The Catholic University of Korea, Seoul, Republic of Korea

## Abstract

Although morphological changes in microglia have been reported to be associated with diabetic retinopathy, little is known about the early changes in the microglia and macrophages during the progression of this condition. The present study was aimed at characterizing retinal microglial activation in the early stages of experimental diabetic retinopathy. Toward this end, a model of diabetic retinopathy was generated by intraperitoneally injecting male Sprague-Dawley rats with streptozotocin. No apparent histological changes were observed during the early stages of experimental diabetic retinopathy. However, at 4 to 16 weeks after the onset of diabetes, the retinas from diabetic rats exhibited higher density of microglia than those from age-matched normal controls, with microglial density peaking at 12 weeks. In particular, the proportion of the activated microglia increased significantly in the diabetic rats, specifically in the nerve fiber and ganglion cell layers, whereas it decreased in the inner plexiform layer within 12 weeks. Furthermore, the resident retinal microglial cells were activated immediately after diabetes induction, peaked at 12 weeks, and remained for up to 16 weeks after disease onset. Thus, experimental diabetic retinopathy causes gradual hypoxia and neuroinflammation, followed by the activation of microglia and the migration of macrophages. The distribution and density of retinal microglial activation changed typically with the progression of the disease in early-stage diabetic rats.

## 1. Introduction

Diabetic retinopathy (DR) is a major complication of diabetes and a leading cause of blindness, with the severe form of the disease affecting the working-age population on a global scale [1, 2]. Microvascular lesions and inflammation play a crucial role in the pathogenesis of DR. Current accepted pharmacological treatments for DR including diabetic macular edema are intravitreal antivascular endothelial growth factor (VEGF) or steroids [3, 4]. They are administered by intravitreal injection and stabilize the detrimental effects of VEGF on microvascular proliferation and permeability [5].

However, there is a considerable unmet need for proper treatment in the early stages of DR. In recent years, many researchers focused on potential pharmacological targets for early diagnosis and treatment of DR. Some microRNAs (miRNAs) can regulate gene expression and related signaling pathways. Lazzara et al. reported a dysregulation in the expression of several miRNAs in diabetic mice and displayed their ability to be potent mediators in the pathological mechanisms associated with DR [6, 7]. These can reveal miRNA-gene-pathways that are modulated in the early phase of DR and other microvascular diseases.

Other studies have reported that pathological changes are induced in the retinal neurovascular units prior to vascular injury [8, 9]. Furthermore, microglia are activated and play a pivotal role in DR. Retinal glial activation and neuronal injury have also been reported during the early stages of DR.

Many studies have suggested that microglial activation could be reflected in neuroinflammatory changes. In diabetic rats, morphological changes in microglia have been reported to occur before neuronal apoptosis and activation of other glial cells in the retina [10, 11]. In a model of streptozotocin- (STZ-) induced diabetes, the expression of retinal Iba-1 was increased by approximately 25%–70%, with a significant difference observed 2 months after diabetes onset [12–14]. Retinal microglial density has been shown to markedly increase in 4-month-old diabetic rats [15].

Although the role of microglia in DR is generally accepted, little is known about the early changes in microglia and macrophages during the progression of DR before chronic damage by ischemia; additionally, chronological changes in the retinal microglia during the early stages of diabetes have not been reported. In the present study, we investigated microglial activation and proliferation during the early course of experimental DR, i.e., before the induction of chronic ischemic damage—to obtain insights into the contribution of these phenomena to DR—using appropriate cell markers.

## 2. Materials and Methods

### 2.1. Animals

Male Sprague-Dawley rats (8 weeks old; weighing 250–300 g; Orient Bio Co., Seongnam-si, Gyeonggi-do, Korea) were used in this study. The animals were kept in a plastic cage in a climate-controlled laboratory with a 12 h light/dark cycle. All procedures performed in studies involving animals were in accordance with the ethical standards of the Institutional Animal Care and Use Committee (IACUC) and Department of Laboratory Animals (DOLA) in the Catholic University of Korea where the studies were conducted. The Catholic University of Korea Songeui Campus accredited the Korea Excellence Animal Laboratory Facility from the Korea Food and Drug Administration in 2017 and acquired Association for Assessment and Accreditation of Laboratory Animal Care (AAALAC) International full accreditation in 2018.

All the animal procedures were carried out in accordance with the Laboratory Animals Welfare Act, Guide for the Care and Use of Laboratory Animals, and Guidelines and Policies for Rodent Experiments provided by IACUC in the School of Medicine, the Catholic University of Korea (Approval number: CUMS-2019-0199-06). This article does not contain any studies with human participants performed by any of the authors.

### 2.2. Induction of Diabetes

A model of diabetes mellitus (DM) was established by a single intraperitoneal injection of STZ (Sigma-Aldrich; 60 mg/kg body weight) in 0.05 M HCl-sodium citrate buffer solution (pH 5.5). The day of STZ injection was defined as day one. The animals were placed in a gas chamber containing 2% isoflurane in oxygen. When unconscious, the animals were removed from the chamber but kept under anesthesia with a mask (1.5% isoflurane in oxygen).

Serum glucose was measured from the tail vein using an automated Accu-Chek glucometer (Roche Diagnostics Ltd., Indianapolis, IN, USA) 3 days following diabetes induction. When serum glucose measured >250 mg/dL on day 3, the development of DM was confirmed, and the rats were used for further experiments. Body weight and serum glucose levels were recorded every week after DM induction.

### 2.3. Tissue Preparation and Histologic Evaluation

The eyeballs were enucleated under anesthesia with zolazepam and xylazine in an aseptic manner. The posterior halves of the globes were immersed in 4% paraformaldehyde in 0.1 M phosphate buffer, pH 7.4. Whole retinas were dissected and immersed in the same fixative for 2 h. After fixation, the retinas were immersed in 30% sucrose, refrigerated overnight, and then flash-frozen in liquid nitrogen and stored at -70°C for preservation. Five-micrometer-thick sections were stained with hematoxylin and eosin (HE).

### 2.4. Immunofluorescence Staining

Retinal pieces were trimmed from the central portion of the superior quadrant and rinsed with 0.01 M phosphate-buffered saline (PBS), pH 7.4. After thorough rinsing, the retinal pieces were embedded in 4% agar and cut into 40 *μ*m thick vertical sections. The retinal sections were collected in culture wells and processed using immunofluorescent microscopy. To block nonspecific binding sites, the sections were treated with buffer B (1% BSA, 0.2% bovine gelatin, and 0.05% saponin in 0.01 M PBS) for 3 h on ice. The sections were then incubated with the following antibodies: monoclonal mouse anti-Ki67 (Thermo Fisher Scientific, Waltham, MA, USA; dilution 1 : 1500), monoclonal mouse anti-ED1 (Bio-Rad, Hercules, California, USA; dilution 1 : 100), and polyclonal rabbit anti-Iba1 (Wako, Japan; dilution 1 : 500) overnight at 4°C. After washing with 0.01 M PBS, immunoreactivity was visualized using secondary antibodies including the species-appropriate Cy3-conjugated donkey anti-rabbit IgG (Jackson ImmunoResearch, West Grove, PA, USA; dilution 1 : 3000) and Alexa Fluor 488 donkey anti-mouse conjugated IgG (Life technologies, Grand Island, NY, USA; dilution 1 : 2000) for 2 h at room temperature. Before mounting, cell nuclei were counterstained with 4′,6-diamidine-2′-phenylindole dihydrochloride (DAPI, Thermo Scientific, Waltham, MA, USA; dilution 1 : 1000). After washing in 0.1 M phosphate buffer, the sections were mounted on a glass slide with a mounting medium (Dako, Santa Clara, CA, USA).

### 2.5. Confocal Microscopy

Immunofluorescent staining was evaluated via confocal laser scanning microscopy (LSM 800 Meta, Carl Zeiss Co. Ltd., Germany). Fluorescent images were captured with red (red: excitation 650 nm, emission 647–700 nm) at 240x, and PEDF images were observed at 400x magnification power. Captured images were converted to JPEG format.

### 2.6. Statistical Analysis

Statistical analysis for comparison of age-matched control and diabetic groups was performed using ANOVA. In individual age groups, Student's *t*-test with Bonferroni adjustment was used to evaluate the significance of the changes between the age-matched controls and diabetic rats.

## 3. Results

### 3.1. Blood Glucose Level and Body Weight of Rats after STZ Injection

**Figure 1:**
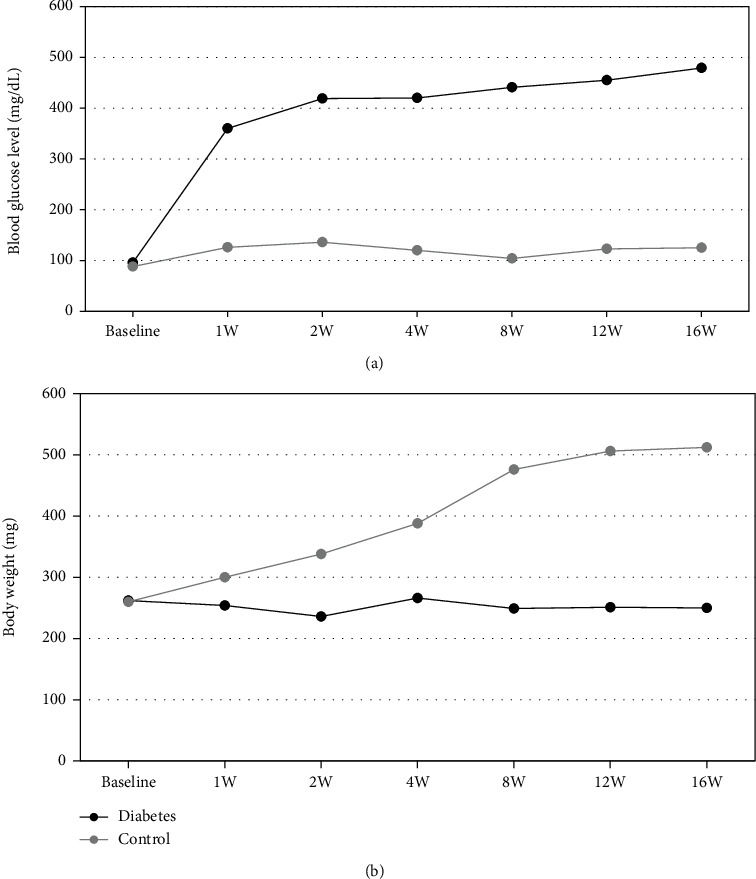
(a) Blood glucose levels (mg/dL) and (b) body weight of control and diabetic rats.

In normal control rats, the blood glucose level remained relatively constant (88 ± 12 mg/dL) for the entire experimental period, and the body weight gradually increased with time (Figure 1). However, in rats injected with STZ, the blood glucose levels increased significantly 1 week after the injection and reached a plateau at 16 weeks (479.6 ± 92.8 mg/dL). At 16 weeks, no significant differences were observed in body weight between STZ-injected rats and controls.

### 3.2. Microglial Morphology

**Figure 2:**
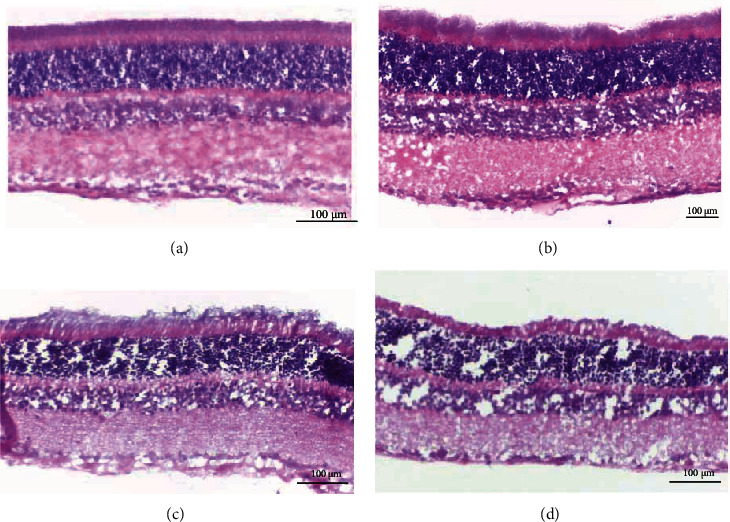
Histology of HE-stained sections in diabetic rats: (a) control; (b) 4 weeks; (c) 8 weeks; (d) 16 weeks. After 8 and 16 weeks, mild thinning of the inner layers of the retina was observed.

Histological analysis of HE-stained sections revealed no significant changes in the retina at early time points. After 8 weeks, mild thinning of the inner layers of the retina was observed (Figure 2). In the control group, Iba-1+ cells were mainly distributed in the inner layers of the retina, and most microglia exhibited a ramified morphology. In the diabetic group, amoeboid morphology was observed in the ganglion cell layer (GCL) and inner plexiform layer (IPL) of the retina, 4 weeks after diabetes onset, with increasing cell number over time. Some microglia, identified as activated microglia, exhibited relatively hypertrophied cell bodies. Activated microglia in the retina of 12- and 16-week diabetic rats presented more hypertrophied cell bodies with coarse processes.

### 3.3. Microglial Density and Activation

**Figure 3:**
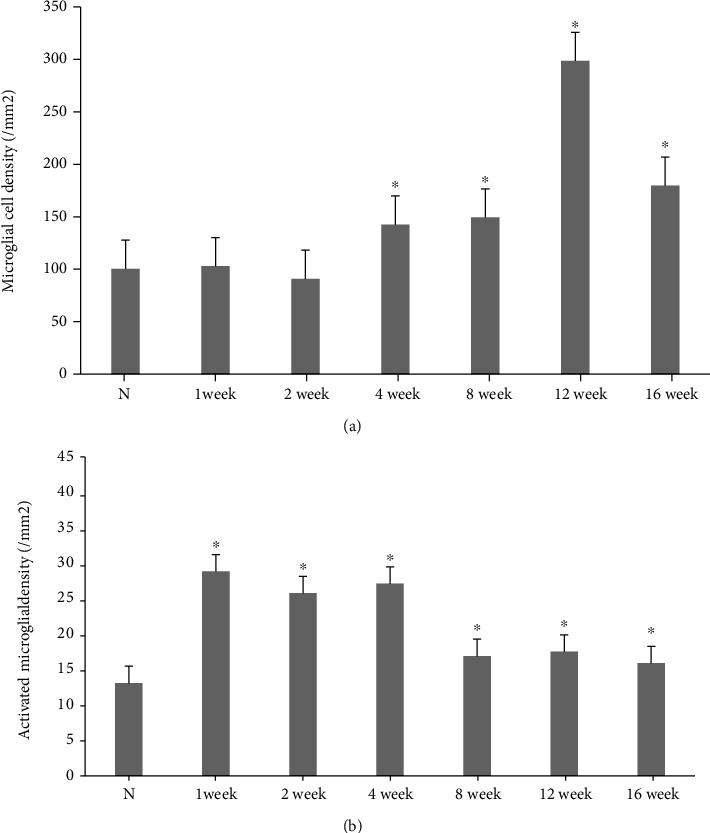
Density of total microglia (a) and activated microglia (b) in the retinas of control and diabetic rats. ^∗^*p* < 0.05 versus control.

The density of Iba-1+ retinal microglia significantly increased in diabetic rats compared to control rats at 4-week postdiabetes onset and peaked at 12-week postdiabetes onset (297.2% ± 69.74%). This was maintained at a high level at 16-week postdiabetes onset (178.9% ± 83.65%) (Figure 3).

**Figure 4:**
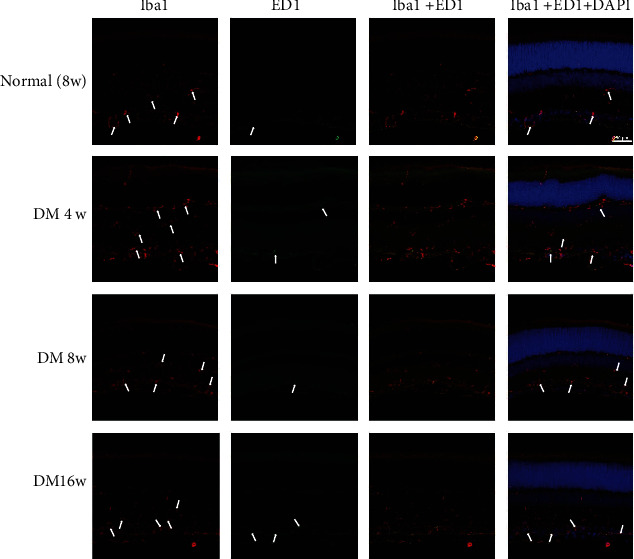
Changes in microglia in normal control and experimental diabetic rat retinas. The retinas were immunostained with Iba-1 antibody (red) and ED1 (green). The nuclei were counterstained with DAPI (blue).

Confocal analysis of double-stained tissue sections confirmed that ED1 expression mostly appeared in Iba1+ cell bodies and it was also observed in some processes. While identifying activated microglia, a significant increase in ED1 expression was detected in the cells exposed to high glucose for 1 week. ED1 expression reached a peak at 4-week postdiabetes onset and remained high in 16-week diabetic rats (Figure 4).

### 3.4. Microglial Distribution

**Figure 5:**
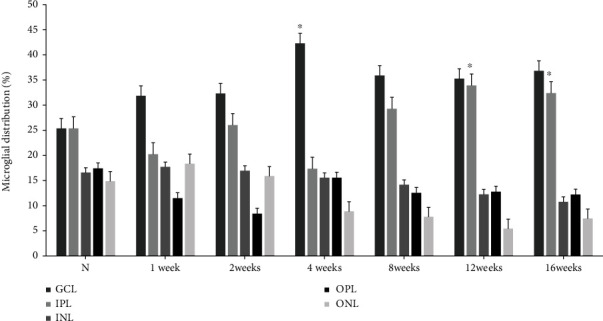
Retinal distribution of microglia in the retinas of control and experimental diabetic rats. In the retinas of 12-week diabetic rats, the proportion of microglia increased in the GCL with a concomitant change in the IPL compared with that in other groups. ^∗^*p* < 0.05 versus other groups.

Microglia in the retinas of control rats were distributed generally in the GCL (25.9% ± 2.47%) and IPL (26.3% ± 1.63%). Moreover, fewer microglia were observed in the outer plexiform layer (OPL), outer nuclear layer (ONL), and rod/cone layer. In 4-week diabetic rats, 45.36 ± 11.42% of microglia were located in the GCL and 20.47 ± 1.34% in the IPL (*p* = 0.025). During this period, the number of microglial cells increased in the GCL, with a concomitant decrease in that in the IPL. However, the distribution of microglial cells changed in the GCL with a simultaneous increase in IPL after 12 and 16 weeks (*p* = 0.046 and *p* = 0.012, respectively) (Figure 5).

### 3.5. Microglial Proliferation

**Figure 6:**
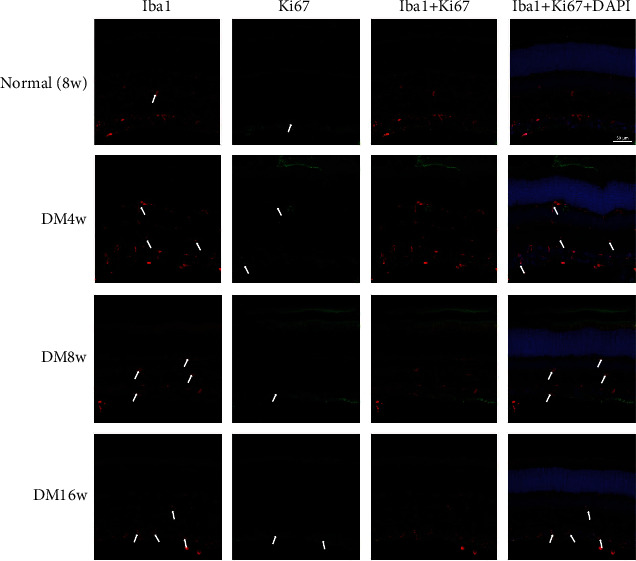
Changes in microglia in normal control and experimental diabetic rat retinas. The retina was immunostained with Iba-1 antibody (red) and Ki-67 (green). The nuclei were counterstained with DAPI (blue).

To determine whether diabetic modeling affects the proliferation of retinal cells, we evaluated the expression of Ki-67, a proliferation cell marker using IHC. In control eyes, weak Ki-67 expression was observed only in the endothelial cells; however, in diabetic rats, weak (but evident) Ki-67 expression was observed mainly in the inner retinal layers. We identified cells that coexpressed Iba-1 and Ki-67, indicating that the active microglial cells (Iba-1+) were also proliferating (Ki-67+) and migrating. The intensity of Iba-1+/Ki-67+ cells increased slightly in 4-week diabetic rats compared to the age-matched control rats (2.35 ± 0.89, *p* > 0.05). However, a significant increase was observed in the 12- and 16-week diabetic rats (3.78 ± 0.54, *p* < 0.05; 3.48 ± 0.61, *p* < 0.05, respectively) (Figure 6).

## 4. Discussion

Recent evidence suggests that the activation and migration of microglial cells may have detrimental and/or beneficial effects on the adjacent neurons. Therefore, it is important to study the mechanisms underlying microglial activation. Insights gained from such studies would enable us to find effective treatment for diseases associated with microglial dysfunction.

Microglial responses to neural damage can be attributed to factors that induce acute neuronal death, recruit peripheral immune cells, and induce the transformation of resident microglia into phagocytic macrophage-like cells. These cells play a pivotal role in the inflammatory processes in the ischemic retina that are normally sequestered from the systemic immune system by the blood–ocular barrier [16, 17].

In human eyes, numerous clusters of hypertrophic microglia can be seen at different stages of DR [18]. A previous study reported microglial activation in the retinas of 4-week diabetic animals, as evidenced by the transition of the microglia from the ramified form to amoeba-shaped form [15, 19]; however, Chen et al. [19] reported that 12-week diabetic rats exhibited a significant increase in the percentage of activated microglia, without any concurrent increase in the microglial density. In the present study, we assessed the microglial responses under proinflammatory conditions in the early stages of STZ-induced diabetes including within 4 weeks.

Iba-1 is a marker of both quiescent and activated microglia. Activated microglia exhibit high Iba-1 expression, enlarged soma, and fewer and shorter processes. In previous studies, Iba-1 was prominently expressed in 4-week diabetes rats, and Iba-1 expression peaked at 12-week postdiabetes onset, indicating a remarkable change in the microglial cells. However, Shi et al. reported that Iba-1 cannot be used on its own to detect active microglia in experimental DR [20]; they suggest that costaining with Iba-1 and other microglial activation markers such as CD11b could accurately reflect the status of microglial activation. Therefore, in the present study, we used ED1 as a marker to evaluate the activation of microglia. IHC revealed that the number of ED1+ cells significantly increased 1 week after diabetes induction; furthermore, activated microglia exhibited hyperplasia and hypertrophy. In our studies, the expression of Iba-1 increased at 4-week postdiabetes onset and peaked at 12 weeks; this result is consistent with that of previous studies [20, 21].

In the present study, although no significant increase in Iba-1 expression was detected, microglial activation—with morphological changes and distribution—was initiated within 1 week of diabetes onset. The majority of microglia was distributed in the inner retina, particularly the IPL, in normal control rats. In experimental diabetic rats, the microglia reacted within 1 week of migratory response. The retinal microglia were redistributed, with higher counts in the GCL and lower counts in the IPL. This change could be relatively attributed to microglial migration, which can be identified under both physiological and pathological conditions [22–24].

In the present study, Iba-1 expression in the retina increased slightly at 4-week postdiabetes onset and reached a significantly higher level at 8- and 12-week postdiabetes onset. The most remarkable change in microglia was detected at 12-week postdiabetes onset; this change was consistent with Ki67 expression. Ki-67 antigen is a well-established marker of proliferating cells. Dual staining for Iba-1 and Ki-67 can enable the detection of active microglia that are proliferating as well. We characterized the proliferating cells in diabetic rats with predominant microglia cells and fewer infiltrating macrophages [25, 26]. The marked increase in the expression of Ki-67 and Iba-1 after the induction of experimental diabetes can presumably be attributed to the infiltration or proliferation of nonresident microglia and macrophages. This hypothesis can be further substantiated by the fact that the expression of Ki-67 and Iba-1 was not detectable in the control eyes, even though blood-borne macrophages were visible, thereby indicating subsequent cellular infiltration. Nevertheless, further studies need to address whether increase in the Ki-67 and Iba-1 expression is because of the infiltration of microglial cells into the retina or is it the result of the proliferation of resident microglial cells.

The principle finding from this longitudinal study was that classical microglial activation was initiated within 1 week of diabetes onset. Even if the total amount of microglia activation did not increase, changes in the distribution of microglia were already initiated within 1 week. Moreover, IHC for Iba-1 and ED1 expression confirmed microglial activation during early stages of experimental diabetes in the retina of rats. Upregulation of these molecules after the induction of diabetes in rats coincides with microglial activation that is implicated in various diseases [24, 27].

Although microglial activation has been recognized as a notable neuropathological change in DR, its role in pathogenesis remains to be elucidated. Activated microglia release various proinflammatory chemokines and cytokines, including tumor necrosis factor-*α*, vascular endothelial growth factor, and interleukin-1*β* [28–30] In recent studies, minocycline (microglial activation inhibitor) has been employed to explore the role of microglia in different diseases [19, 31]. However, their results could merely be a restrictive effect. More precise research is needed to investigate the function and influence of microglia.

## 5. Conclusions

Microglia were activated in the retinas of early-stage diabetic rats; changes were observed in microglial morphology, density, and distribution within 1 week of diabetes onset. Early activation of retinal microglia in diabetic rats may be indicative of their importance in DR pathogenesis, which requires further investigation.

## Data Availability

The data used to support the findings of this study are available from the corresponding author upon request.
